# Effects of a 7-week active breaks intervention program on physical literacy and body mass index

**DOI:** 10.3389/fpsyg.2025.1535729

**Published:** 2025-02-10

**Authors:** Javier Urbano-Mairena, Laura Muñoz-Bermejo, Jorge Carlos-Vivas, Raquel Pastor-Cisneros, José Adrián Montenegro-Espinosa, María Mendoza-Muñoz

**Affiliations:** ^1^Social Impact and Innovation in Health (InHEALTH), University Centre of Mérida, University of Extremadura, Merida, Spain; ^2^Physical Activity for Education, Performance and Health (PAEPH) Research Group, Faculty of Sport Sciences, University of Extremadura, Cáceres, Spain; ^3^Promoting a Healthy Society Research Group (PHeSO), Faculty of Sport Sciences, University of Extremadura, Caceres, Spain; ^4^One Health Research Group, Universidad de Las Américas, Quito, Ecuador; ^5^Physical and Health Literacy and Health-Related Quality of Life (PHYQoL), Faculty of Sport Science, University of Extremadura, Cáceres, Spain

**Keywords:** physical literacy, active breaks, schoolchildren, CAPL-2, physical activity

## Abstract

**Introduction:**

Physical literacy (PL) emerges as a highly effective resource for creating lasting physical activity habits at a time when physical inactivity has become one of the main risk factors in our population. Thus, active breaks (AB) could be an ideal option for increasing physical activity time in schoolchildren.

**Methods:**

A cross-sectional quasi-experimental study was conducted, involving 89 participants aged 8-12 years from Extremadura. The Canadian assessment of physical literacy (CAPL-2) was administered to assess the participants' physical literacy level. The BMI was also measured.

**Results:**

Higher scores were obtained in all domains and subdomains of the CAPL in the experimental group. Significant differences were found both in total physical literacy score (*p* < 0.001) and in all domains and subdomains, except for the subdomains self-reported question, intrinsic motivation and knowledge and understanding domain (*p* = 0.344).

**Conclusion:**

The results of the study support the positive effects of an AB program of PL and its domains. The development of active break programs could help to increase the PL level of schoolchildren.

## 1 Introduction

Physical inactivity has become the main risk factor in our population (Rezende et al., 2016; Bull et al., 2020) due to its contribution to the development of cardiovascular disease (Je et al., 2013), obesity, overweight, and diabetes (Bull et al., 2020; Michel et al., 2022). Despite this, the minimum physical activity (PA) guidelines and recommendations are clear that young people should engage in at least 60 min of moderate and vigorous physical activity per day, yet only than 20% of adolescents comply with these recommendations (WHO, 2021).

The promotion of healthy lifelong lifestyles justifies the use of resources to motivate and raise awareness of the reasons for and purposes for being more active (Frohlich and Potvin, 2008). Therefore, all interventions aimed at promoting PA should focus not only on the amount and intensity of physical activity (Cairney et al., 2019), but also on raising participants' awareness of why it is important to be active and to maintain sustainable PA habits over time. Therefore, changes in behavior and PA habits will provide a healthier lifestyle, reducing levels of sedentary lifestyles in childhood and adolescence (Pate et al., 2011; Chaput et al., 2020), as well as diseases associated with physical inactivity, such as obesity, diabetes, or mental illness, with PA as the main tool to achieve this (van Sluijs et al., 2021). The importance of all this is heightened by the knowledge that children's elementary school years are a critical period for the development of long-term healthy lifestyles (Conti and Heckman, 2013), as habits formed in childhood are more likely to persist into adulthood (Pérez-Navero et al., 2018).

Physical literacy (PL) is emerging as a highly useful tool not only for promoting long-lasting habits of PA over time (Borchers and Pieler, 2010), but also for understanding why young people are or are not physically active (Whitehead, 2010). While physical activity is defined as “any bodily movement produced by the contraction of skeletal muscles that increases energy expenditure above the resting metabolic rate” (Caspersen et al., 1985, p. 127), PL is different and refers to an individual's understanding, knowledge, physical competence, motivation, and ability to be physically active on a sustained basis over time (Whitehead, 2010).

Nevertheless, there in undoubtedly a need to increase the amount of PA time for young people. Given its importance, active breaks (AB) could be an excellent option to increase children's physical activity during breaks from learning tasks (Howie et al., 2014), providing intention and commitment to physical activity during these periods (Bailey et al., 2024). This type of practice has increased through several strategies (Howie et al., 2014), promoting not only physical but also psychological outcomes (Erwin et al., 2014). Furthermore, a recent review revealed that ~14% of PA interventions for children in Europe are based on AB (Porter et al., 2024). These interventions are mostly delivered during AB between classes, allowing them to be more active (McLellan et al., 2022). On the other hand, carrying out these activities during breaks in the school day (AB) could make a valuable contribution to promoting the practice of PA among schoolchildren (Hyndman, 2017).

Thus, the PA programs implemented during this period of time have shown how they help students to become more physically active, increasing not only the levels of physical activity but also the intensity with which the practice is performed (Ansón and García-Jiménez, 2017).

Currently, several studies have revealed how PL interventions have had positive effects on PL, both in extracurricular activities (Mandigo et al., 2019), and in physical education classes (Coyne et al., 2019). Studies on PL interventions during recess are scarce, but some studies implementing PA-based interventions have shown positive effects on PL (Mendoza-Munoz et al., 2022). Although no studies have been found that directly relate the positive effects of AB to PL, benefits of AB on academic performance (Petrigna et al., 2022), attention (Méndez-Giménez and Pallasá-Manteca, 2023), and concentration (Contreras Jordan et al., 2020; Fiorilli et al., 2021), wellbeing and PA habits (Petrigna et al., 2022; Peiris et al., 2022) have been reported. Thus, PL work during these periods could be highly beneficial for improving the domains of motivation (Méndez-Giménez and Pallasá-Manteca, 2023), physical competence, and daily physical activity time (Galle et al., 2020; Masini et al., 2020a).

Therefore, the aim of this study was to evaluate the effect of a 7-week AB intervention on PL and its domains in schoolchildren aged 8–12 years and to examine the body composition of participants before and after the intervention.

## 2 Material and methods

### 2.1 Study design

A cross-sectional quasi-experimental study with pre and post intervention assessments was designed to evaluate the effect of a 7-week PL based active breaks program.

### 2.2 Ethics

The research received the approval of the Bioethics and Biosafety Committee at the University of Extremadura (registral number: 91/2024), in accordance with the revisions made to the Helsinki Declaration by the 64th General Assembly of the World Medical Association (Fortaleza, Brazil, 2013) and in compliance with Law 14/2007 on Biomedical Research.

### 2.3 Procedures

Participants aged 8–12 years from three primary schools were recruited. The management team of the schools was contacted by e-mail and in person. To this end, the objectives and procedures of the study, the participants required, the test be conducted, and the duration and content of the intervention were explained to those responsible for each of the schools. The schools contacted the parents or guardians of the students, who decided whether or not their children would participate in the study. Once contact was established, authorization was obtained from the pupils' parents or guardians, and informed consent was obtained from the pupils themselves.

Prior to the start of the study, of the two classes selected from each center, one was randomly assigned to the experimental group (EG) and the other as the control group (CG). Both the assessments and the intervention were conducted by qualified personnel who were part of the study; school personnel did not participate in the study. Children in the CG only participated in the initial and final assessments. Participants in the CG performed free activities as in any normal recess. The children in the EG participated in the assessments and also in a program of active breaks based on physical literacy development for 7 weeks.

### 2.4 Participants

The final sample consisted of 89 participants, of which 49.4% were male and 50.6% were female. To be included in this study, the students needed to meet the following inclusion criteria: (1) age 8–12 years; (2) no pathology preventing physical activity; (3) informed consent from parents or legal guardians; (4) residence in Extremadura.

### 2.5 Intervention

This intervention was based on the study protocol developed by Mendoza-Muñoz et al. (2022). The theoretical and conceptual underpinning of this intervention focused on physical literacy, covering four distinct areas: physical competence, daily physical activity, knowledge and understanding, and motivation and confidence. The study lasted a total of 9 week, with the first and ninth weeks corresponding to the pre and post intervention assessment, respectively. During the remaining 7 weeks, the EG preformed a total of 21 physical literacy-based sessions, lasting 20 min, during the break period of the school day. On the other hand, the CG conducted the activities that the normally conducted in their daily lives during the school day, with the same timetable, frequency, and duration as the EG.

The sessions were divided into two distinct parts. The first part was aimed at working on knowledge content in an active way, through different activities such as orienteering races, relay races, or linking the answer (Table 1), where we work on content such as healthy lifestyle habits, benefits of physical activity, how to be more active, sports and their values, and basic physical capacities. On the other hand, during the second half of the session, a activity was played that remained constant throughout each week's sessions, but whose difficulty was progressively adjusted in each session, achieving a gradual increase in challenge during the week. This activity and its different variants were aimed at increasing the physical competence, motivation and confidence of the participants. The sports and games they participated in were *Mother Earth defender, Protect the pinnie, Keep it up, Farmers Shepherding Sheep, Tail chase, Switch it up, Knock Down*. These activities were obtained from PlaySport (Ontario Physical Health Education Association, 2022), initially developed by Ontario Physical and Health Education Association in partnership with Brock University. Each part lasted ~10 min, and each of the content was covered during the same week. The resources used were developed by the research team (Supplementary material S1).

**Table 1:** Activities and contents developed during the AB program.

	**First part of session**		**Second part of session** ^*^	
	**(10 min per day)**		**(10 min per day)**	
**Week**	**Activities**	**Activities Content**	**Activities**	**Objective**
1	Physical literacy and body composition assessment			
2	Orientation relay	Physical lifestyle habits	Mother earth defender	Enhance physical competence, motivation and confidence
3	Orientation relay	Benefits of PA How to be more active?	Protect the pinnie	
4	Relay races	Sports skills	Keep it up	
5	Relay races	Type of sports	Farmers Shepherding sheep	
6	Link the answer	Kind of fitness (strength and endurance)	Tail chase	
7	Link the answer	Kind of fitness (speed and flexibility)	Switch it up	
8	Link the answer	All contents	Knock down	
9	Physical literacy and body composition assessment			

^*^All the activities belonging to the second part of the session were obtained from PlaySport (Association), initially developed by Ontario Physical and Health Education Association in partnership with Brock University.

For the development of the intervention and the presentation of results, the PLIRT guidelines established by Carl et al. (2023) for PL interventions have been considered (Supplementary Table S1).

### 2.6 Measures

In order to carry out the procedures, the study protocol Wellbeing, Obesity and Motricity Observatory (WOMO) (Mendoza-Munoz et al., 2020) was followed. The evaluation guide Canadian Assessment of Physical Literacy 2 (CAPL-2), developed by the Healthy Active Living and Obesity Research Group (HALO), belonging to the Children's Hospital of Eastern Ontario Research Institute (Heatlhy Active Living Obesity Research Group, 2017) in its Spanish adaptation was used for the assessment of the PL.

#### 2.6.1 Anthropometry

Although no standardized data were used for the measurements, the conditions established by the OMS (de Onis et al., 2007) and the ALADINO study (Perez-Farinos et al., 2013) were followed. For the bodyweight measurement of participants' were measured without shoes and dressed in light clothing. A bioimpedancemeter (Tanita MC-780 MA, Tanita Corporation, Tokyo, Japan) was used. Weight was recorded in kg. Height was obtained using a height gauge (Tanita Tantois, Tanita Corporation, Tokyo, Japan), recorded in centimeters and approximation in millimeters. Participants stood with arms relaxed and feet balanced on a vertical surface, perpendicular to the ground.

To obtain the body mass index (BMI), the sex, age, and height of each participant were entered into the electrical bioimpedance device.

#### 2.6.2 Physical literacy

The Canadian Assessment of Physical Literacy 2 (CAPL-2) was used for the assessment of the PL (Tremblay et al., 2018; Health, Economy, Motricity and Education Research Group (HEME) and Promoting a Healthy Society Research Group, PHSO; Longmuir et al., 2018a). The scores ranges from 0 to 100 points, resulted from the sum of its four domains. Each domain is composed of different test, obtained a final score of each of them. The domains of PL are: physical competence, daily physical activity behaviors, knowledge and understanding and motivation and confidence.

1. Physical competence domain (PC domain). It allows to evaluate the physical competence of the participants. This domain consists of three tests, each one evaluated from 1 to 10 points, obtaining a final score for this domain out of 30 points. These tests are:
  a) Isometric abdominal plank during 2 min (Longmuir et al., 2018b).
  b) Progressive aerobic cardiovascular endurance run “PACER.” Capacity cardiorespiratory test.
  c) Canadian Agility and Movement Skill Assessment “CAMSA.” Agility circuit.
2. Daily physical activity behavior (DB domain). The final score of this domain is obtained from the number of total daily steps of the participant recorded through an activity wristband (Xiaomi mi Band 3, Xiaomi Corporation, Pekin, China), and the number of minutes of physical activity performed by the participants for at least 60 min.
3. Knowledge and understanding (K&U domain). To obtain the score for this domain, participants answer five questions, scoring up to 10 points. Four of the five question scored 0 or 1 each, while the last question is a fill in the blanks question in a story, scored from 1 to 6 points.
4. Motivation and confidence (M&C domain). This domain attempts to measure participants' confidence and motivation to be physically active. Its score ranges from 1 to 30 points. It consists of four parts: intrinsic motivation, competence, predilection and adequacy.

### 2.7 Statistical analysis

Statistical procedures and calculations were performed using Statistical Package for the Social Sciences (SPSS, version 25.0; IBM SPSS Inc., Armonk, NY, USA). Data are presented as mean and standard deviation (SD) or median and interquartile range (IR) for variables with normal and non-normal distributions, respectively. Shapiro-Wilk and Levene's tests were used to test the normality and homogeneity of the data. Then, inferential tests were performed for all dependent variables. A two-way repeated measures analysis of variance (ANOVA) was performed to examine the interaction between two factors: group (experimental and control) and two timepoints (baseline, post-intervention) in all dependent variables. Significant differences were set at *p* ≤ 0.05.

## 3 Results

The characteristics of the participants in both the control and experimental groups are shown in Table 2. No significant differences were found between the control and experimental groups for any of the anthropometric variables or for age (*p* = 0.168; *p* = 0.508).

**Table 2:** Total sample characteristics and stratified by group.

	**All participants**	**Experimental group (*****n*** = **43)**		**Control group (*****n*** = **46)**		**Between-group comparison**
**Gender**		**Male**	**Female**	**Male**	**Female**	
*N* (%)	89 (100)	21 (48.8)	22 (51.2)	23 (50)	23 (50)	
	**Median (IR)**	**Median (IR)**		**Median (IR)**		***p***
Age (years)	11 (2)	11 (2)		10 (2)		0.508
Weight (kg)	39.60 (43.3)	42.8 (43.3)		39.15 (29.3)		0.209
BMI (kg/m^2^)	18.85 (17.01)	19.02 (15.73)		18.40 (14.6)		0.426
	**Mean (SD)**	**Mean (SD)**		**Mean (SD)**		
Height (cm)	146.18 (8.30)	147.47 (9.31)		144.98 (7.50)		0.168

Table 3 shows the BMI and CAPL-2 scores for both the control and experimental groups, as well as the intergroup and intragroup differences.

**Table 3:** Outcome of BMI and physical literacy measures at baseline and post-intervention.

	**Experimental group (*****n*** = **43)**					**Control group (*****n*** = **46)**						
	**Baseline**		**Post-intervention**		**Within-group**	**Baseline**		**Post-intervention**		**Within-group**	**Between group pre**	**Between group post**
	**Mean**	**SD**	**Mean**	**SD**	***p***	**Mean**	**SD**	**Mean**	**SD**	***p***	***p***	***p***
BMI	19.82	4.00	19.76	4.01	0.667	18.93	3.44	18.97	3.56	0.763	0.260	0.330
DB domain (points)	17.070	1.117	19.814	1.104	< 0.001	19.304	1.080	19.370	1.067	0.927	0.154	0.773
Self-reported question (points)	3.581	0.208	3.907	0.164	0.085	3.587	0.201	4.065	0.158	0.010	0.985	0.489
Diary steps (points)	13.488	1.032	15.907	1.033	0.001	15.717	0.998	15.304	0.999	0.545	0.124	0.676
PC domain (points)	15.821	0.885	19.360	0.828	< 0.001	17.261	0.856	18.458	0.800	0.032	0.245	0.435
CAMSA (points)	5.332	0.268	6.919	0.238	< 0.001	6.522	0.259	6.871	0.230	0.099	0.002	0.886
Plank (points)	6.907	0.509	7.837	0.433	0.025	6.804	0.492	7.413	0.419	0.126	0.885	0.483
PACER (points)	3.581	0.310	4.605	0.373	< 0.001	3.935	0.300	4.174	0.361	0.348	0.415	0.409
M&C domain (points)	25.912	0.432	27.421	0.464	0.003	25.630	0.418	25.000	0.448	0.196	0.641	< 0.001
Predilection (points)	6.784	0.167	7.233	0.167	0.020	6.889	0.162	6.546	0.162	0.063	0.652	0.004
Adequacy (points)	6.814	0.192	7.374	0.154	0.016	6.524	0.185	6.270	0.149	0.253	0.279	< 0.001
Intrinsic motivation (points)	6.453	0.151	6.535	0.146	0.617	6.293	0.146	6.283	0.141	0.945	0.448	0.217
Competence (points)	5.860	0.158	6.279	0.148	0.013	5.924	0.153	5.902	0.144	0.892	0.773	0.071
K&U domain (points)	6.535	0.263	6.837	0.233	0.344	6.587	0.254	6.957	0.226	0.232	0.887	0.714
Overall physical literacy (points)	65.337	1.899	73.433	1.814	< 0.001	68.783	1.836	69.784	1.754	0.350	0.195	0.152

BMI, Body mass index; DB, daily behavior domain; PC, physical competence domain; CAMSA, Canadian Agility and Movement Skill Assessment; PACER, Progressive Aerobic Cardiovascular Endurance Run; M&C, motivation and confidence domain; K&U, knowledge and understanding domain.

Regarding the experimental group, all domain and subdomain scores of the CAPL were higher in the post-intervention assessment than pre-intervention. Significant im-provements were found in total PL (*p* < 0.001), as well as in all domains and subdomains except self-reported question (*p* = 0.085), Intrinsic motivation (*p* = 0.617) and K&U domain (*p* = 0.344).

About intergroup differences, the results reported no differences between the control and experimental groups in the pretest, except for the CAMSA test, where the score was higher in the control group than in the experimental group (*p* = 0.002). In the case of the post-test, significant improvements were detected in the experimental group with respect to the control, in the M&C domain and its subdomain's predilection and adequacy.

No intergroup or intragroup differences were reported for BMI.

## 4 Discussion

The concept of PL has become a highly relevant and widely researched concept in recent years (Gilic et al., 2022), and has become a fundamental concept for schoolchildren's participation in a wider range of physical activities (Caldwell et al., 2020). Currently, several studies of PA programs have reported changes in PL levels of participants. These studies were conducted during Physical Education classes (Kriellaars et al., 2019) as well as during after-school activities (Mandigo et al., 2019; Bremer et al., 2020). However, only one study protocol was found that addressed the effects of an active breaks program on physical literacy, on which this study is based (Mendoza-Munoz et al., 2022).

The present study aimed to evaluate the effects of a 7-week active breaks intervention on PL and its domains in schoolchildren aged 8–12 years, also analyzing participants' body composition before and after the intervention. The results obtained reveal that the program was effective in improving general PL and in several specific domains, except for the K&U domain (*p* = 0.334), in which the EG scored higher than the CG, although without significant differences.

Activity during active breaks in the school day has been shown to be an excellent way to improve the health of schoolchildren, both in terms of reducing body fat, increasing speed and coordination (Aguilar-Jurado et al., 2020), as well as improving cognitive performance (Latorre-Román et al., 2021). In this sense, Vicedo et al. (2021) concluded that these periods represent valuable opportunities to increase children's physical activity time and help them reach the minimum recommended levels of healthy PA.

Because of its importance, it is inevitable to think about how AB could be used to improve health and, with it, the time dedicated to physical activity of schoolchildren. If we review systematic studies on AB (Vicedo et al., 2021), we find numerous interventions aimed at promoting PA, however, those that address the importance of AB to improve PL are less frequent. Promoting PL is critical, given its impact on health. Nevertheless, when considering the benefits of AB interventions, one could find and ideal tool and space to help schoolchildren adop a healthy lifestyle through PA and PL.

Although there are currently different tools that can assess the PL in schoolchildren, Shearer et al. (2021), in their systematic review of PL assessment tools, highlighted that the CAPL-2 is currently the most robust explicit assessment instrument at present, demonstrating validity and reliability in the assessment of PL. Thus, several studies have attempted to give visibility to the assessment of PL in schoolchildren in different countries through the CAPL-2 (Dania et al., 2020; Mendoza-Munoz et al., 2024; Hadier et al., 2024; Elsborg et al., 2021; Knisel et al., 2024; Li et al., 2020; Longmuir et al., 2015), showing that the level of PL in this population is low or “in progress” (Li et al., 2020; Longmuir et al., 2015; Dania et al., 2020).

Analyzing the evolution of each of the domains in both groups after the intervention, we can observe that in the DB domain, significant differences were found in the EG (*p* < 0.001), but not in one of its subdomains, despite obtaining a higher score in the post-assessment (Self-reported question; *p* > 0.05). Our results differ from those found by Mendoza-Munoz et al. (2022) who, in their 4-week study, found no significant improvements in this domain, which could be due to the duration of the intervention or to the contents of the intervention itself. On the other hand, Masini et al. (2020b), in their 14-week study of active breaks, showed significant improvements in the number of weekly steps taken by their participants. Similarly, Torrandell and Vidal-Conti (2021) found that those schoolchildren who were more active during breaks had a greater number of hours of physical activity during the week.

Therefore, the EG in our study may have increased significantly in this domain after receiving more encouragement to engage in daily physical activity, relative to the CG, and the duration of the intervention may be entirely relevant in empowering and stimulating participants sufficiently to increase their daily and weekly physical activity time. The fact that significant differences were found in a more objective test such as the number of steps participants took per week, compared to a more subjective test such as answering a self-reported question about the number of days they were physically active, may support the idea that the intervention time was sufficient to motivate students to increase their daily steps, but not to make them aware that by doing the activities during breaks, they were simultaneously increasing the number of days they were physically active. Future interventions based on AB should further investigate the long-term effects on participants's daily physical activity routines.

On the other hand, the EG obtained significant improvements in the PC domain and its subdomains, in contrast to the CG, which did not obtain significant improvements in any of the subdomain but did obtain significant improvements in the final score of the domain. One possible explanation for this could be that each of the subdomains achieves higher scores, though not sufficient to yield significant improvements individually. Therefore, these improvements might have accumulated to influence the overall score of the domain, resulting in a significant change in the global computation.

The significant differences found in the EG in this domain may be due to the relationship between PL, health, and fitness (Caldwell et al., 2020). In this way, PL becomes a determining factor in the development of children's physical fitness (Gilic et al., 2022). Increasing pupils' motor engagement time during active breaks, through activities that promote the development of cardiorespiratory capacity, strength, and agility may be fundamental for their development. In this regard, Mendoza-Munoz et al. (2022) reported significant improvements in this domain, as well as in the CAMSA and PACER subdomains. These results, similar to those observed in our study, could also have been influenced by the association demonstrated in various studies between the general level of PL and cardiorespiratory capacity (Lang et al., 2018) and agility (Mandigo et al., 2019). Thus, the significant improvements in the general level of PL might have contributed to advancements in this domain and its subdomain.

Regarding the M&C domain, our results show significant differences in the total score of the domain and its subdomains for the EG. However, in the subdomain of intrinsic motivation, no significant differences were found for this group (*p* = 0.617). Furthermore, a significant increase was also observed in the EG compared to CG after the intervention. Therefore, this domain could become the most relevant factor for children to reach the minimum recommendations for physical activity (Belanger et al., 2018), since both the motivation of students to be active and the place of activity have a positive influence on increasing their activity (Lang et al., 2018). In this regard, several physical activity-based interventions (Abós et al., 2016), and PL (Bremer et al., 2020) have reported improvements in motivation level, reflected in a willingness to participate in a greater number of physical activities and sports after their interventions (Bremer et al., 2020).

No significant improvements were found in the knowledge and understanding domain, although a positive trend was observed in the EG. The literature has shown that knowledge about physical activity is an essential component of the cognitive domain of PL (Gilic et al., 2022), although its direct effects on physical activity practice are still unknown. However, several studies have shown positive relationships of this domain with PC (Li et al., 2020) and M&C (Li et al., 2020; Knisel et al., 2024). Due to the influence of PA on the children's cognitive performance (Reloba et al., 2016), it could be that due to the low intensity of the task proposed during the intervention, or the short time spent performing PA daily, no significant differences were found in this domain (Luque-Illanes et al., 2021). In addition, the limitations of the intervention may have been insufficient to facilitate understanding and retention of the knowledge necessary for children to recognize the value of physical activity in their lives. This opens up a line of future research where future studies could investigate the importance of knowledge and understanding of physical activity and its practice, as well as explore whether the intervention time is limited to achieve meaningful learning.

In terms of body composition, no significant differences in BMI were found in either group, highlighting the importance of a longer intervention duration to observe substantial changes in this variable (Henaghan et al., 2008). However, given that several studies have reported differences in students' PL levels as a function of BMI (Delisle Nystrom et al., 2018; Mendoza-Munoz et al., 2021), further research should study the long-term effects of physical literacy on schoolchildren and explore the benefits of physical literacy on schoolchildren's body composition.

One of the main strengths of this study is that it allows us to know and support the findings found by Mendoza-Munoz et al. (2022) about the benefits of implementing active breaks programs on PL in schoolchildren. However, the main difference with respect to that study is not only the duration, but also in the type of activities and content addressed throughout the intervention. In addition, the use of the CAPL-2 to measure the PL in schoolchildren allows it to be assessed in a comprehensive and objective manner in its different domains. Therefore, the findings of this study have important applications for health promotion, physical education, and educational policy. The implementation of this type of program could help not only to improve PL, but also to know the level of PL of schoolchildren, making it possible to take measures in the different intervention programs by adapting them to the needs of all students, since a child with a low level of PL will tend to avoid physical activity as much as possible, will have little confidence in their physical capacity and will not be motivated to participate in structured physical activities (Tremblay et al., 2018), therefore, physical literacy in all schoolchildren will be fundamental.

### 4.1 Limitations

The main strength of this study is the use of an objective assessment of physical literacy, validated for assessment in schoolchildren between 8 and 12 years of age (Longmuir et al., 2018a). However, this study has several methodological limitations that need to be carefully considered. Firstly, the sample size (*n* = 89) is relatively small for a study of this nature, which could affect the statistical power and consequently the detection of significant effects, especially in those subdomains where no significant differences were found (self-reported questions, intrinsic motivation and knowledge and understanding domain). This limitation becomes particularly relevant when analyzing the effects in specific age subgroups within the studied range (8–12 years). The selection of the sample by convenience, limited to schools in Extremadura, is another substantial limitation that affects the external validity of the study. The specific characteristics of the Extremadura context, including its socio-economic, cultural and educational particularities, could differ significantly from other Spanish regions or international contexts. This geographical and contextual specificity limits the generalizability of the results to other school populations with different socio-demographic characteristics, educational resources or pre-existing physical activity programmes. The duration of the active-break intervention represents another significant limitation. Although positive effects were found on the total physical literacy score and in most domains and subdomains, the intervention period was not long enough to assess the sustainability of these effects in the medium and long term. This temporal limitation is particularly relevant in the context of physical literacy, where the main goal is to create lasting physical activity habits. The absence of longitudinal follow-up prevents us from determining whether the observed gains in physical literacy levels are maintained beyond the immediate intervention period or whether these changes actually translate into sustainable physical activity habits.

## 5 Conclusions

The results of this study support the positive effects on physical literacy and its domains of an activity-based active breaks program covering all domains. In contrast to other studies, this work was notable for the duration and specific focus of the activities and for the application of the CAPL-2 for a comprehensive assessment of physical literacy domains. These findings have important implications for the design of intervention programs and the formulation of educational policies that promote physical literacy and health in schoolchildren. The implementation of active breaks could contribute to improving the level of PL in students, which is crucial to prevent physical inactivity in those with low confidence and motivation to engage in structured physical activity.

In conclusion, active breaks programs specifically designed to improve physical literacy offer a promising avenue for strengthening physical health and engagement in physical activity in schoolchildren. Future studies could focus on interventions of longer duration and in different contexts to explore the long-term effects on children's physical behavior and body composition, thus facilitating the adoption of active and healthy lifestyles from an early age.

## Data Availability

The original contributions presented in the study are included in the article/Supplementary material, further inquiries can be directed to the corresponding authors.

## References

[B1] Abós A. Sevil J. Julián J. A. Abarca-Sos A. García-González L. (2016). Improving students' predisposition towards physical education by optimizing their motivational processes in an acrosport unit. Euro. Phys. Educ. Rev. 23, 444–460. 10.1177/1356336X16654390

[B2] Aguilar-Jurado M. A. Gil-Madrona P. Ortega-Dato J. F. Rodríguez-Blanco Ó. F. (2020). Mejora de la condición física y la salud en estudiantes tras un programa de descansos activos. Rev. Española Salud Públ. 92:e201809068. Available at: https://scielo.isciii.es/pdf/resp/v92/1135-5727-resp-92-e201809068.pdf PMC11587305 30178781

[B3] Ansón A. B. García-Jiménez J. V. (2017). Niveles de actividad física durante los recreos escolares: revisión teórica. EmásF Rev. digital educación física 8, 12–26. Available at: https://dialnet.unirioja.es/servlet/articulo?codigo=5963357

[B4] Bailey R. P. Payne R. Raya-Demidoff A. Samsudin N. Scheuer C. (2024). Active recess: school break time as a setting for physical activity promotion in European primary schools. Health Educ. J. 83, 531–543. 10.1177/00178969241254187

[B5] Belanger K. Barnes J. D. Longmuir P. E. Anderson K. D. Bruner B. Copeland J. L.. (2018). The relationship between physical literacy scores and adherence to Canadian physical activity and sedentary behaviour guidelines. BMC Public Health 18:1042. 10.1186/s12889-018-5897-4 30285783 PMC6167767

[B6] Borchers A. Pieler T. (2010). Programming pluripotent precursor cells derived from Xenopus embryos to generate specific tissues and organs. Genes 1, 413–426. 10.3390/genes1030413 24710095 PMC3966229

[B7] Bremer E. Graham J. D. Cairney J. (2020). Outcomes and feasibility of a 12-week physical literacy intervention for children in an afterschool program. Int. J. Environ. Res. Public Health 17:3129. 10.3390/ijerph17093129 32365870 PMC7246927

[B8] Bull F. C. Al-Ansari S. S. Biddle S. Borodulin K. Buman M. P. Cardon G.. (2020). World Health Organization 2020 guidelines on physical activity and sedentary behaviour. Br. J Sports Med. 54, 1451–1462. 10.1136/bjsports-2020-102955 33239350 PMC7719906

[B9] Cairney J. Dudley D. Kwan M. Bulten R. Kriellaars D. (2019). Physical literacy, physical activity and health: toward an evidence-informed conceptual model. Sports Med. 49, 371–383. 10.1007/s40279-019-01063-3 30747375

[B10] Caldwell H. A. T. Di Cristofaro N. A. Cairney J. Bray S. R. MacDonald M. J. Timmons B. W. (2020). Physical literacy, physical activity, and health indicators in school-age children. Int. J. Environ. Res. Public Health 17:5367. 10.3390/ijerph17155367 32722472 PMC7432049

[B11] Carl J. Barratt J. Arbour-Nicitopoulos K. P. Barnett L. M. Dudley D. A. Holler P.. (2023). Development, explanation, and presentation of the physical literacy interventions reporting template (PLIRT). Int. J. Behav. Nutr. Phys. Act 20:21. 10.1186/s12966-023-01423-3 36805731 PMC9938627

[B12] Caspersen C. J. Powell K. E. Christenson G. M. (1985). Physical activity, exercise and physical fitness: definitions and distinctions for helath-related reserach. Public Health Rep. 100, 126–131. 3920711 PMC1424733

[B13] Chaput J. P. Willumsen J. Bull F. Chou R. Ekelund U. Firth J.. (2020). 2020 WHO guidelines on physical activity and sedentary behaviour for children and adolescents aged 5-17 years: summary of the evidence. Int. J. Behav. Nutr. Phys. Act 17:141. 10.1186/s12966-020-01037-z 33239009 PMC7691077

[B14] Conti G. Heckman J. J. (2013). The developmental approach to child and adult health. Pediatrics 131(Suppl. 2), S133–S141. 10.1542/peds.2013-0252d 23547057 PMC4075134

[B15] Contreras Jordan O. R. Infantes-Paniagua A. Prieto-Ayuso A. (2020). Effects of active breaks in the attention and concentration of the elementary school students. Rev. Interuniv. Form. Profr. 95, 145–160. Available at: https://www.redalyc.org/journal/274/27467982009/29998131

[B16] Coyne P. Vandenborn E. Santarossa S. Milne M. M. Milne K. J. Woodruff S. J.. (2019). Physical literacy improves with the Run Jump Throw Wheel program among students in grades 4-6 in southwestern Ontario. Appl. Physiol. Nutr. Metab. 44, 645–649. 10.1139/apnm-2018-0495 31032623

[B17] Dania A. Kaioglou V. Venetsanou F. (2020). Validation of the Canadian assessment of physical literacy for Greek children: understanding assessment in response to culture and pedagogy. Euro. Phys. Educ. Rev. 26, 903–919. 10.1177/1356336X20904079

[B18] de Onis M. Onyango A. W. Borghi E. Siyam A. Nishida C. Siekmann J. (2007). Development of a WHO growth reference for school-aged children and adolescents. Bull. World Health Organ. 85, 660–667. 10.2471/BLT.07.043497 18026621 PMC2636412

[B19] Delisle Nystrom C. Traversy G. Barnes J. D. Chaput J. P. Longmuir P. E. Tremblay M. S.. (2018). Associations between domains of physical literacy by weight status in 8- to 12-year-old Canadian children. BMC Public Health 18:1043. 10.1186/s12889-018-5898-3 30285688 PMC6167768

[B20] Elsborg P. Melby P. S. Kurtzhals M. Tremblay M. S. Nielsen G. Bentsen P.. (2021). Translation and validation of the Canadian assessment of physical literacy-2 in a Danish sample. BMC Public Health 21:2236. 10.1186/s12889-021-12301-7 34886833 PMC8656017

[B21] Erwin H. E. Ickes M. Ahn S. Fedewa A. (2014). Impact of recess interventions on children's physical activity–a meta-analysis. Am. J. Health Promot. 28, 159–167. 10.4278/ajhp.120926-LIT-470 23875990

[B22] Fiorilli G. Buonsenso A. Di Martino G. D. Crova C. Centorbi M. Grazioli E.. (2021). Impact of active breaks in the classroom on mathematical performance and attention in elementary school children. Healthcare 9:1689. 10.3390/healthcare9121689 34946415 PMC8701340

[B23] Frohlich K. L. Potvin L. (2008). Transcending the known in public health practice: the inequality paradox: the population approach and vulnerable populations. Am. J. Health Promot. 98, 216–221. 10.2105/AJPH.2007.114777 18172133 PMC2376882

[B24] Galle F. Pecoraro P. Calella P. Cerullo G. Imoletti M. Mastantuono T.. (2020). Classroom active breaks to increase children's physical activity: a cross-sectional study in the province of Naples, Italy. Int. J. Environ. Res. Public Health 17:6599. 10.3390/ijerph17186599 32927849 PMC7560134

[B25] Gilic B. Malovic P. Sunda M. Maras N. Zenic N. (2022). Adolescents with higher cognitive and affective domains of physical literacy possess better physical fitness: the importance of developing the concept of physical literacy in high schools. Children 9:796. 10.3390/children9060796 35740733 PMC9221622

[B26] Hadier S. G. Liu Y. Long L. Hamdani S. M. Z. H. Khurram H. Hamdani S. D.. (2024). Assessment of physical literacy in 8- to 12-year-old Pakistani school children: reliability and cross-validation of the Canadian assessment of physical literacy-2 (CAPL-2) in South Punjab, Pakistan. BMC Public Health 24:1726. 10.1186/s12889-024-19185-3 38943131 PMC11212239

[B27] Health Economy, Motricity and Education Research Group (HEME) and Promoting a Healthy Society Research Group (PHSO). Manual Para la Administración de CAPL-2 (Evaluación Canadiense de Alfabetización Física). Versión Española. Available at: https://www.activehealthykids.org/wp-content/uploads/2022/04/capl-manual-es.pdf (accessed May 2, 2024).

[B28] Heatlhy Active Living and Obesity Research Group (2017). CAPL-2 Manual, 2nd Edn. Healthy Active Living and Obesity Research Group. Available at: https://www.activehealthykids.org/wp-content/uploads/2022/04/capl-2-manual-en.pdf

[B29] Henaghan J. McWhannell N. Foweather L. Cable N. T. Batterham A. M. Stratton G.. (2008). The effect of structured exercise classes and a lifestyle intervention on cardiovascular risk factors in primary schoolchildren: an exploratory trial (the A-CLASS project). Pediatric Exerc. Sci. 20, 169–180. 10.1123/pes.20.2.169 18579898

[B30] Howie E. K. Beets M. W. Pate R. R. (2014). Acute classroom exercise breaks improve on-task behavior in 4th and 5th grade students: a dose–response. Mental Health Phys. Activity 7, 65–71. 10.1016/j.mhpa.2014.05.002

[B31] Hyndman B. (2017). Contemporary School Playground Strategies for Healthy Students. Singapore: Springer. 10.1007/978-981-10-4738-1

[B32] Je Y. Jeon J. Y. Giovannucci E. L. Meyerhardt J. A. (2013). Association between physical activity and mortality in colorectal cancer: a meta-analysis of prospective cohort studies. Int. J. Cancer 133, 1905–1913. 10.1002/ijc.28208 23580314

[B33] Knisel E. Bremer M. Nałecz H. Wascher L. Laudańska-Krzemińska I. (2024). Validation of The Canadian assessment of physical literacy – CAPL-2 questionnaire for German and Polish school children. Phys. Cult. Sport Stud. Res. 104, 1–14. 10.2478/pcssr-2024-0014

[B34] Kriellaars D. J. Cairney J. Bortoleto M. A. C. Kiez T. K. M. Dudley S. Aubertin P. (2019). The impact of circus arts instruction in physical education on the physical literacy of children in grades 4 and 5. J. Teach. Phys. Educ. 38, 162–170. 10.1123/jtpe.2018-0269

[B35] Lang J. J. Chaput J. P. Longmuir P. E. Barnes J. D. Belanger K. Tomkinson G. R.. (2018). Cardiorespiratory fitness is associated with physical literacy in a large sample of Canadian children aged 8 to 12 years. BMC Public Health 18, 1–13. 10.1186/s12889-018-5896-5 30285694 PMC6167777

[B36] Latorre-Román P. A. Berrios-Aguayo B. Aragón-Vela J. Pantoja-Vallejo A. (2021). Effects of a 10-week active recess program in school setting on physical fitness, school aptitudes, creativity and cognitive flexibility in elementary school children. A randomised-controlled trial. J. Sports Sci. 39, 1277–1286. 10.1080/02640414.2020.1864985 33407022

[B37] Li M. H. Sum R. K. W. Tremblay M. Sit C. H. P. Ha A. S. C. Wong S. H. S.. (2020). Cross-validation of the Canadian assessment of physical literacy second edition (CAPL-2): the case of a Chinese population. J. Sports Sci. 38, 2850–2857. 10.1080/02640414.2020.1803016 32787646

[B38] Longmuir P. E. Boyer C. Lloyd M. Yang Y. Boiarskaia E. Zhu W.. (2015). The Canadian assessment of physical literacy: methods for children in grades 4 to 6 (8 to 12 years). BMC Public Health 15:767. 10.1186/s12889-015-2106-6 26260572 PMC4532252

[B39] Longmuir P. E. Gunnell K. E. Barnes J. D. Belanger K. Leduc G. Woodruff S. J.. (2018a). Canadian assessment of physical literacy second edition: a streamlined assessment of the capacity for physical activity among children 8 to 12 years of age. BMC Public Health 18:1047. 10.1186/s12889-018-5902-y 30285687 PMC6167760

[B40] Longmuir P. E. Woodruff S. J. Boyer C. Lloyd M. Tremblay M. S. (2018b). Physical literacy knowledge questionnaire: feasibility, validity, and reliability for Canadian children aged 8 to 12 years. BMC Public Health 18:1035. 10.1186/s12889-018-5890-y 30285679 PMC6167766

[B41] Luque-Illanes A. Gálvez-Casas A. Gómez-Escribano L. Escámez-Bañoz J. C. Tárraga-Marcos L. Tárraga-López P.. (2021). ¿Mejora la actividad física el rendimiento académico en escolares? Una revisión bibliográfica. J. Negative No Positive Res. 6, 84–103. 10.19230/jonnpr.3277

[B42] Mandigo J. Lodewyk K. Tredway J. (2019). Examining the impact of a teaching games for understanding approach on the development of physical literacy using the passport for life assessment tool. J. Teach. Phys. Educ. 38, 136–145. 10.1123/jtpe.2018-0028

[B43] Masini A. Marini S. Gori D. Leoni E. Rochira A. Dallolio L.. (2020a). Evaluation of school-based interventions of active breaks in primary schools: a systematic review and meta-analysis. J. Sci. Med. Sport 23, 377–384. 10.1016/j.jsams.2019.10.008 31722840

[B44] Masini A. Marini S. Leoni E. Lorusso G. Toselli S. Tessari A.. (2020b). Active breaks: a Pilot and feasibility study to evaluate the effectiveness of physical activity levels in a school based intervention in an Italian primary school. Int. J. Environ. Res. Public Health 17:4351. 10.3390/ijerph17124351 32560544 PMC7345227

[B45] McLellan G. Arthur R. Donnelly S. Bakshi A. Fairclough S. J. Taylor S. L.. (2022). Feasibility and acceptability of a classroom-based active breaks intervention for 8-12-year-old children. Res. Q. Exerc. Sport 93, 813–824. 10.1080/02701367.2021.1923627 34748469

[B46] Méndez-Giménez A. Pallasá-Manteca M. (2023). Efecto de los descansos activos sobre procesos atencionales y la regulación motivacional en escolares. Apunts Educ. Física Deportes 1, 49–57. 10.5672/apunts.2014-0983.es.(2023/1).151.05

[B47] Mendoza-Munoz M. Adsuar J. C. Perez-Gomez J. Munoz-Bermejo L. Garcia-Gordillo M. A. Carlos-Vivas J.. (2020). Well-being, obesity and motricity observatory in childhood and youth (WOMO): a study protocol. Int. J. Environ. Res. Public Health 17:2129. 10.3390/ijerph17062129 32210073 PMC7143434

[B48] Mendoza-Munoz M. Barrios-Fernandez S. Adsuar J. C. Pastor-Cisneros R. Risco-Gil M. Garcia-Gordillo M. A.. (2021). Influence of body composition on physical literacy in Spanish children. Biology 10:482. 10.3390/biology10060482 34072359 PMC8228674

[B49] Mendoza-Munoz M. Calle-Guisado V. Pastor-Cisneros R. Barrios-Fernandez S. Rojo-Ramos J. Vega-Munoz A.. (2022). Effects of active breaks on physical literacy: a cross-sectional Pilot study in a region of Spain. Int. J. Environ. Res. Public Health 19:7597. 10.3390/ijerph19137597 35805254 PMC9266253

[B50] Mendoza-Muñoz M. Carlos-Vivas J. Villafaina S. Parraca J. A. Vega-Munoz A. Contreras-Barraza N.. (2022). Effects of a physical literacy breaks (PLBreaks) program on physical literacy and body composition in Portuguese schoolchildren: a study protocol. Biology 11:910. 10.3390/biology11060910 35741431 PMC9219803

[B51] Mendoza-Munoz M. Lopez-Gil J. F. Pastor-Cisneros R. Castillo Paredes A. Urbano Mairena J. Tremblay M.. (2024). Cross-validation of the Canadian assessment of physical literacy second edition (CAPL-2) for Spanish children. BMJ Open Sport Exerc. Med. 10:e001971. 10.1136/bmjsem-2024-001971 39006391 PMC11243216

[B52] Michel J. Bernier A. Thompson L. A. (2022). Physical activity in children. JAMA Pediatr. 176:622. 10.1001/jamapediatrics.2022.0477 35467714

[B53] Ontario Physical and Health Education Association (2022). PlaySport. Available at: www.playsport.net (accessed October 25, 2022).

[B54] Pate R. R. Mitchell J. A. Byun W. Dowda M. (2011). Sedentary behaviour in youth. Br. J. Sports Med. 45, 906–913. 10.1136/bjsports-2011-090192 21836174

[B55] Peiris D. L. I. H. K. Duan Y. Vandelanotte C. Liang W. Yang M. Baker J. S.. (2022). Effects of in-classroom physical activity breaks on children's academic performance, cognition, health behaviours and health outcomes. A systematic review and meta-analysis of randomised controlled trials. Int. J. Environ. Res. Public Health 19:9479. 10.3390/ijerph19159479 35954831 PMC9368257

[B56] Perez-Farinos N. Lopez-Sobaler A. M. Dal Re M. A. Villar C. Labrado E. Robledo T.. (2013). The ALADINO study: a national study of prevalence of overweight and obesity in Spanish children in 2011. Biomed. Res. Int. 2013:163687. 10.1155/2013/163687 24089663 PMC3780583

[B57] Pérez-Navero J. L. Tejero-Hernández M. A. Llorente-Cantarero F. J. (2018). Influencia del deporte y la actividad física en la infancia y adolescencia. Vox Paediatr. 25, 49–56. Available at: https://munideporte.com/imagenes/documentacion/ficheros/002DFB2B.pdf

[B58] Petrigna L. Roggio F. Trovato B. Zanghì M. Musumeci G. (2022). Are physically active breaks in school-aged children performed outdoors? A systematic review. Sustainability 14:3713. 10.3390/su14073713

[B59] Porter A. Walker R. House D. Salway R. Dawson S. Ijaz S.. (2024). Physical activity interventions in European primary schools: a scoping review to create a framework for the design of tailored interventions in European countries. Front. Public Health 12:1321167. 10.3389/fpubh.2024.1321167 38389941 PMC10883314

[B60] Reloba S. Chirosa L. J. Reigal R. E. (2016). Relación entre actividad física, procesos cognitivos y rendimiento académico de escolares: revisión de la literatura actual. Rev. Andaluza Medicina Deporte 9, 166–172. 10.1016/j.ramd.2015.05.008

[B61] Rezende L. F. M. Sa T. H. Mielke G. I. Viscondi J. Y. K. Rey-Lopez J. P. Garcia L. M. T.. (2016). All-cause mortality attributable to sitting time: analysis of 54 countries worldwide. Am. J. Prev. Med. 51, 253–263. 10.1016/j.amepre.2016.01.022 27017420

[B62] Shearer C. Goss H. R. Boddy L. M. Knowles Z. R. Durden-Myers E. J. Foweather L.. (2021). Assessments related to the physical, affective and cognitive domains of physical literacy amongst children aged 7-11.9 years: a systematic review. Sports Med. Open 7:37. 10.1186/s40798-021-00324-8 34046703 PMC8160065

[B63] Torrandell M. X. B. Vidal-Conti J. (2021). Relación entre la actividad física durante el recreo escolar, actividad física semanal y expediente académico. Sportis Sci. J. Sch. Sport Phys. Educ. Psychomotr. 7, 150–170. 10.17979/sportis.2021.7.1.6850

[B64] Tremblay M. S. Longmuir P. E. Barnes J. D. Belanger K. Anderson K. D. Bruner B.. (2018). Physical literacy levels of Canadian children aged 8-12 years: descriptive and normative results from the RBC Learn to Play-CAPL project. BMC Public Health 18:1036. 10.1186/s12889-018-5891-x 30285693 PMC6167776

[B65] van Sluijs E. M. F. Ekelund U. Crochemore-Silva I. Guthold R. Ha A. Lubans D.. (2021). Physical activity behaviours in adolescence: current evidence and opportunities for intervention. Lancet 398, 429–442. 10.1016/S0140-6736(21)01259-9 34302767 PMC7612669

[B66] Vicedo J. C. P. Martínez J. M. Polo M. L. Ayuso A. P. (2021). Recreos activos como estrategia de promoción de la actividad física: una revisión sistemática. Retos nuevas tendencias educación física deporte recreación 40, 135–144. 10.47197/retos.v1i40.82102

[B67] Whitehead M. (2010). Physical Literacy: Throughout the Lifecourse. Abingdon: Routledge. 10.4324/9780203881903

[B68] WHO (2021). WHO Guidelines on Physical Activity and Sedentary Behaviour. Ginebra: World Health Organization. 33369898

