# Supplementary material for: Effects of a 7-week active breaks intervention program on physical literacy and body mass index
Source: Front Psychol. 2025 Feb 10;16:1535729. doi: 10.3389/fpsyg.2025.1535729 (PMC11847806; doi:10.3389/fpsyg.2025.1535729)
Supplement: Supplementary file 1 [file Table_1.pdf]

Table S1. The PLIRT Checklist

Ítem	Description	Location in the Paper and/or Comment
**Tittle**		
1	Highlight the role of PL in the Tittle	Page 1
**Background and Definition**		
2	Describe the relevance of PL for the target population/group/individual	Page 2
3	Explain your conceptualization of PL and refer to a holistic definition of PL	Page 2
4	Formulate PL-related goals/aims of your study	Page 2
**Assessment**		
5a	*If quantitative:* Choose multidimensional assessment strategy of PL and provide information about psychometric properties	Page 3
5b	*If qualitative:* Develop a qualitative method that closely aligns with PL theory and the different domains	-
**Design and Content**		
6	Ensure that your interventional approach is in line with PL-compatible philosophical assumptions	No mention
7	Mention the intervention provider(s), describe his/her/their expertise specific to PL, and any specific training given	No mention
8	Report in detail intervention content related to all PL domains	Page 3
9	Explain whether and how you realized the integrative arrangement of content/techniques	
10	Consider general guidelines for intervention reporting	Page 3
**Evaluation**		
11	Describe how the PL intervention was accepted and/or whether it was implemented as intended (modifications, fidelity, compliance, adherence)	Page 3
12a	*If quantitative:* Report transparently how the different PL domains (and if initially intended, other relevant outcomes such as health) were affected by the intervention	Page 6
12b	*If qualitative:* Characterize the strengths, weaknesses, and challenges of your PL intervention; the different PL domains may help you structure the analysis and results	-
**Discussion and Conclusion**		
13	Discuss the limitations of you PL intervention, especially whether (if yes, where and why) you had to deviate meaningfully from your planned conceptualization	Page 7
14	Break down your experiences with the PL intervention and derive sound recommendations for future studies	Page 7

Note: PLIRT Physical Literacy Interventions Reporting Template, PL Physical Literacy
