# Supplementary material for: Effects of a 7-week active breaks intervention program on physical literacy and body mass index
Source: Front Psychol. 2025 Feb 10;16:1535729. doi: 10.3389/fpsyg.2025.1535729 (PMC11847806; doi:10.3389/fpsyg.2025.1535729)
Supplement: Supplementary file 2 [file Presentation_1.pdf]

WEEK 2 - SESSION 1

Orientation Relay

Participants are organized into different groups, all starting from the same point (black marker). Each group is given a map with a single colored marker, which indicates the location of a question. To answer the question, the must use the options provided next to the map. Once they find the question and asnwer it, they return to the starting point to confirm their response and receive the next map. The team that correctly completes all five maps first is declared the winner.

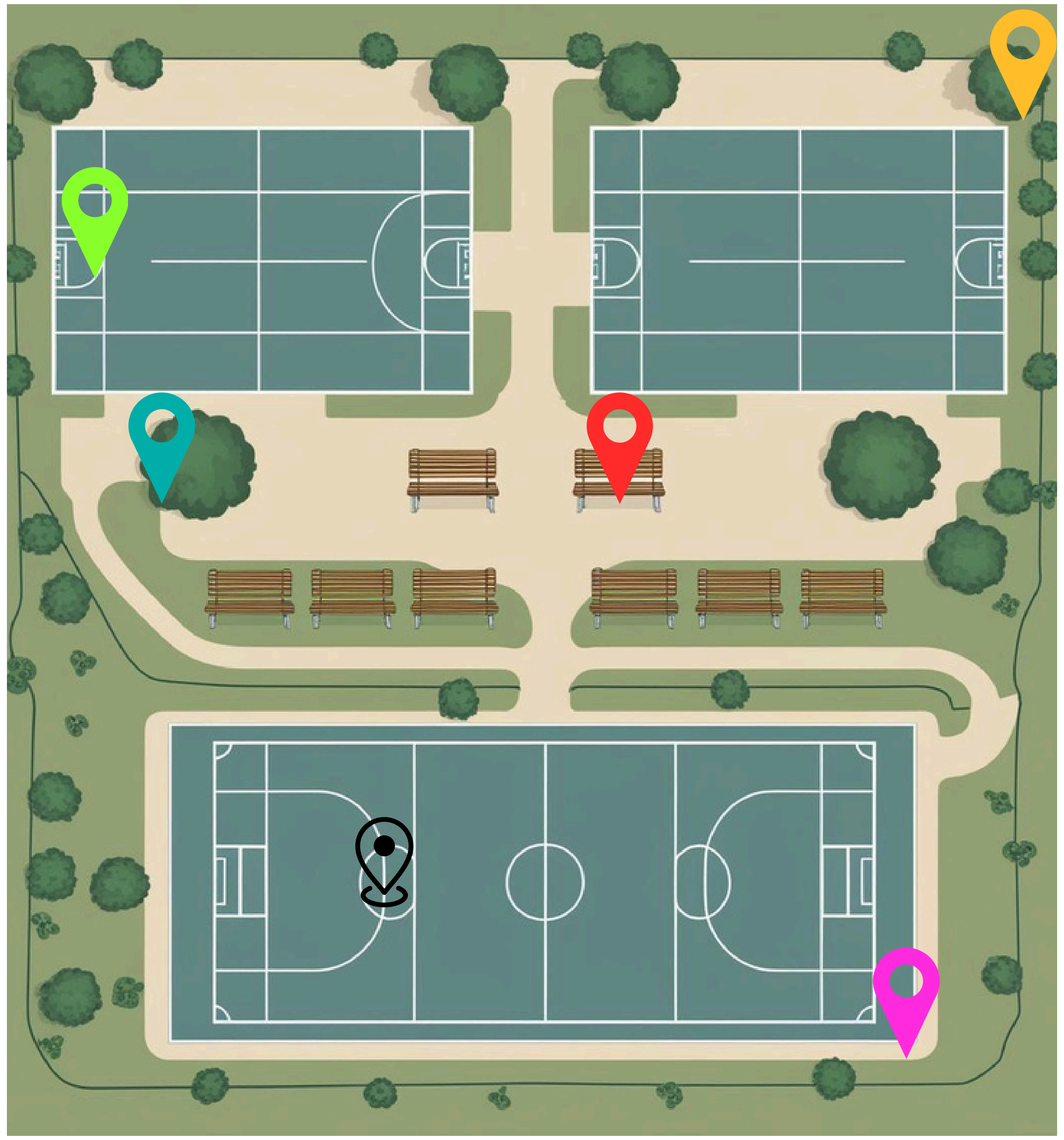

MAP 1

Question number: __
(If I do not engage in regular physical exercise, it may...)

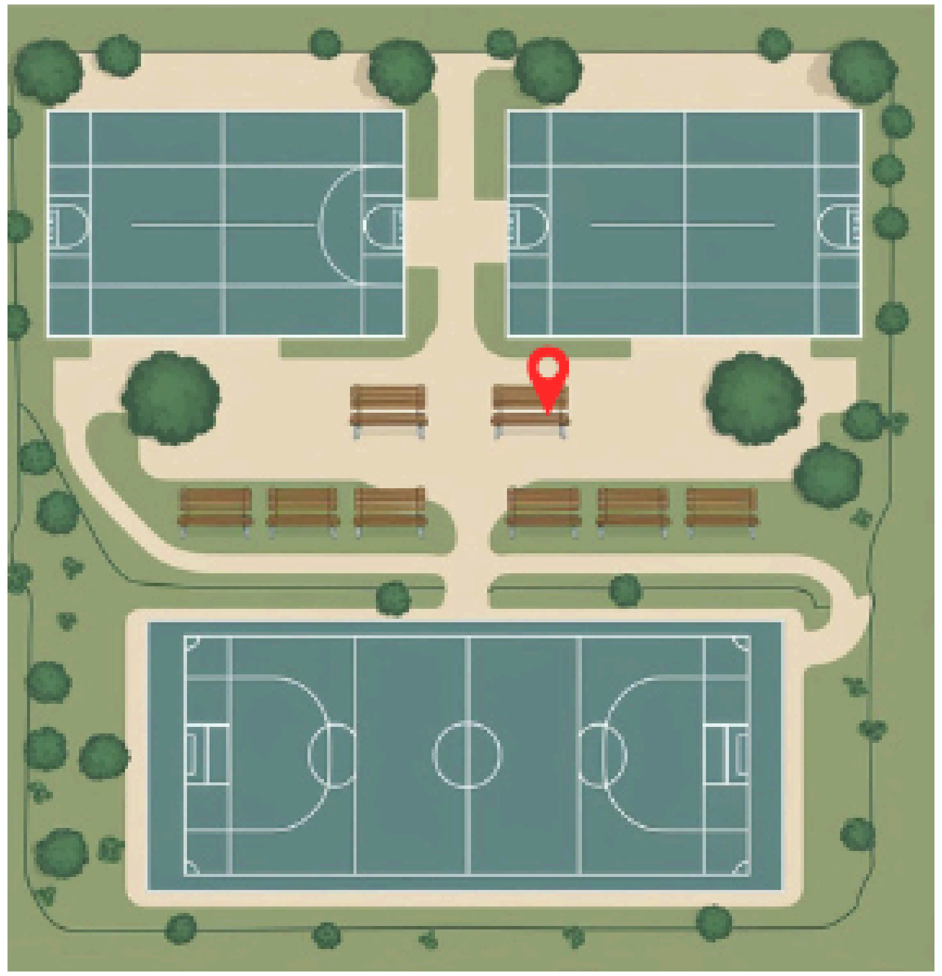

Answers:

Improving my heart's functioning system

Improve the health of my bones

Contract diseases related to physical inactity, such as obesity or heat disease

None of the above is correct

MAP 2

Question number: __

(Children of my age can only use screens...)

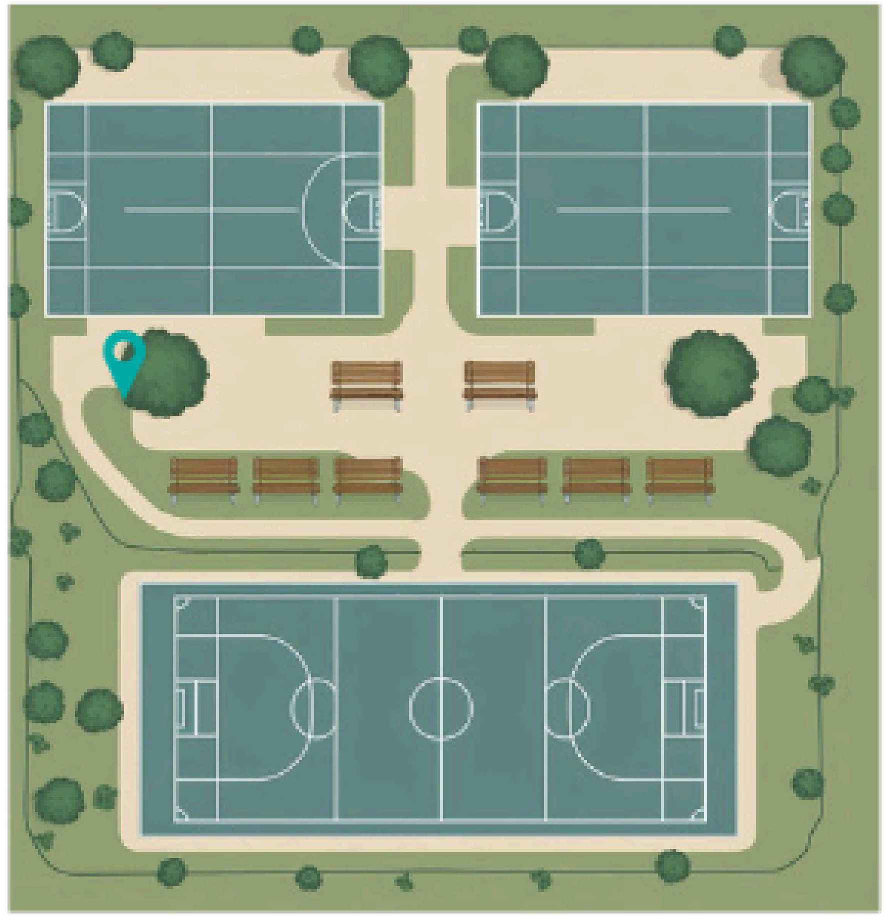

Answers:

- At most two hours per day
- At most one hour a day
- At most 3 hours a day
- Whenever they want to

MAP 3

Question number: __
(Continuous physical exercise can...)

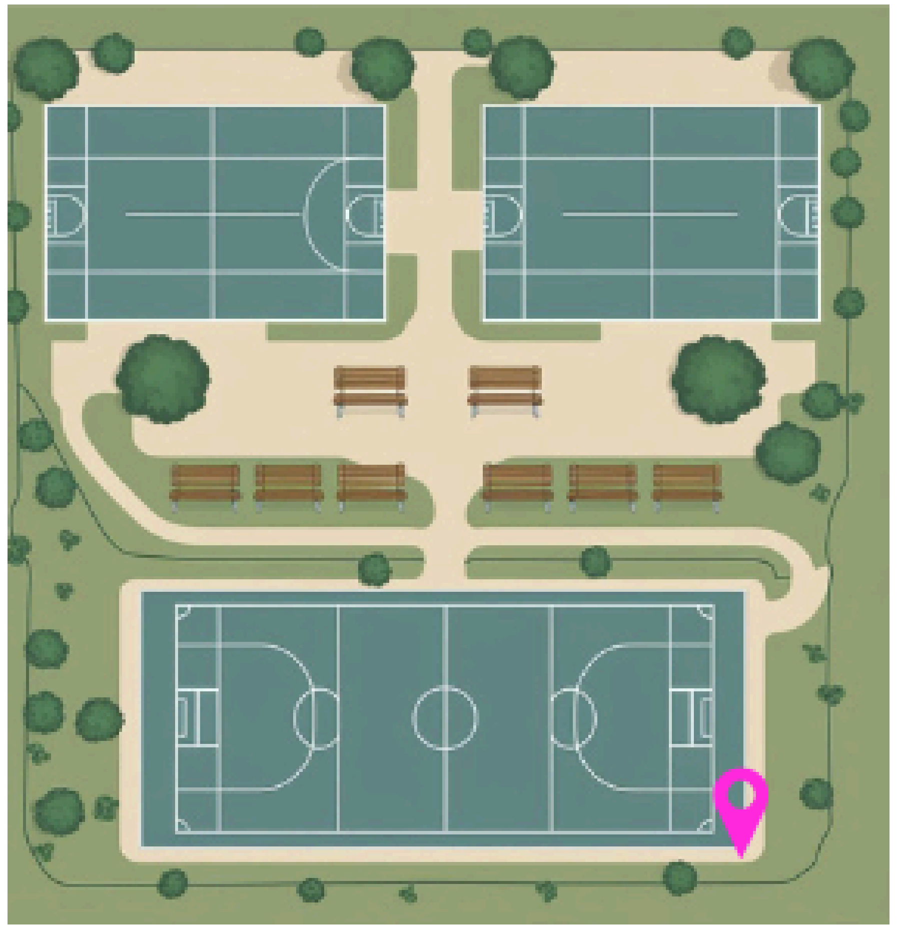

Answers:

- Increase breathing capacity
- Promote the development of my muscles
- Improve my academic results
- All are correct

MAP 4

Question number: __

(What is a sedentary lifestyle?)

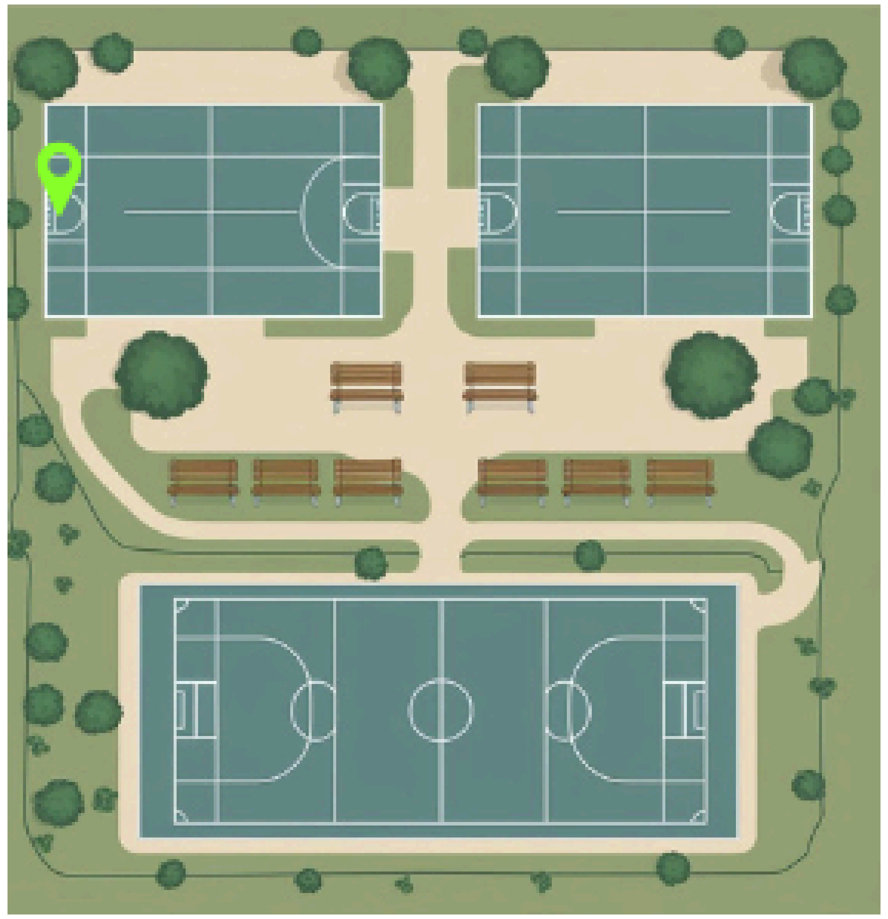

Answers:

A lifestyle that children exhibit when there is a lot of movement and a high level of physical activity

A lifestyle that children exhibit when there is little movement and a low level of physical activity

A lifestyle that children exhibit when there is a lot of movement and a low level of physical activity

A lifestyle that children exhibit when there is alittle movement and a high level of physical activity

MAP 5

Question number: __
(How much sleep should children my age daily to get a good night's rest?)

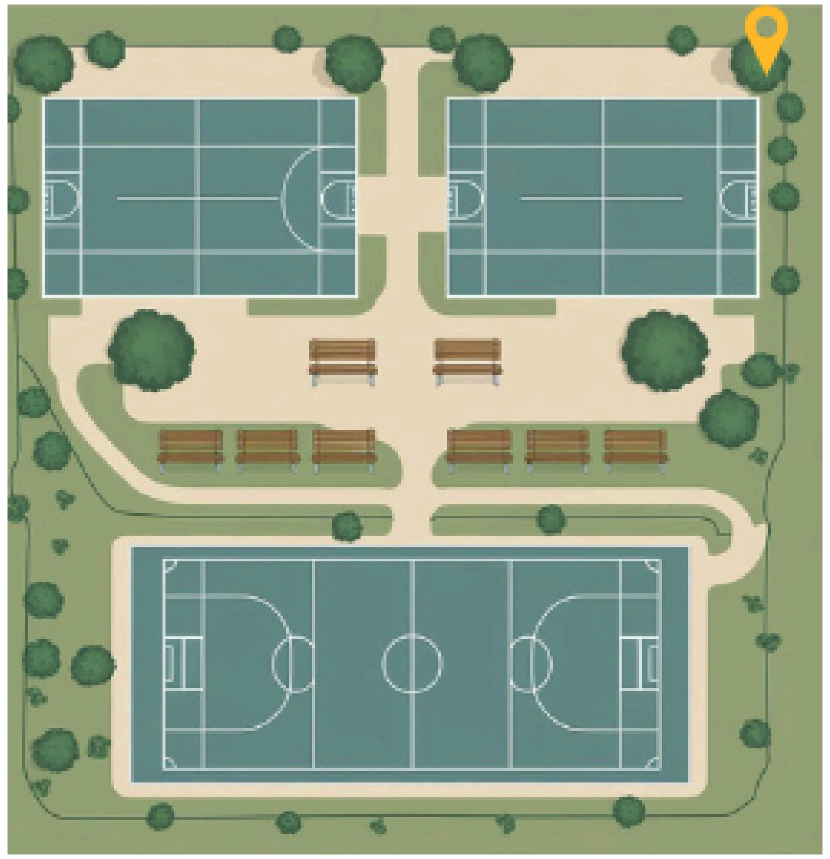

Answers:

More than 12 hours

Between 9 and 12 hours

Between 7 and 8 hours

Less than 8 hous

WEEK 5 - SESSION 2

RELAY RACES

Participants are organized into different groups at a starting point (blue marker). Each group receives a sheet of paper containing six numbered questions. At a distance of 15 meters from the starting point (black marker), a paper displaying pictures of various sport, the answers to the question, is placed.

Participants take turns (one member from each group at a time), to quickly go and look at this sheet to find the answer to one question. They can discuss their findings with the rest of the group before submitting their answer.

Once the group provides an answer, and the instructor confirms it is correct, the participant passes the baton to the next teammate. The first team to answer all the question correctly wins.

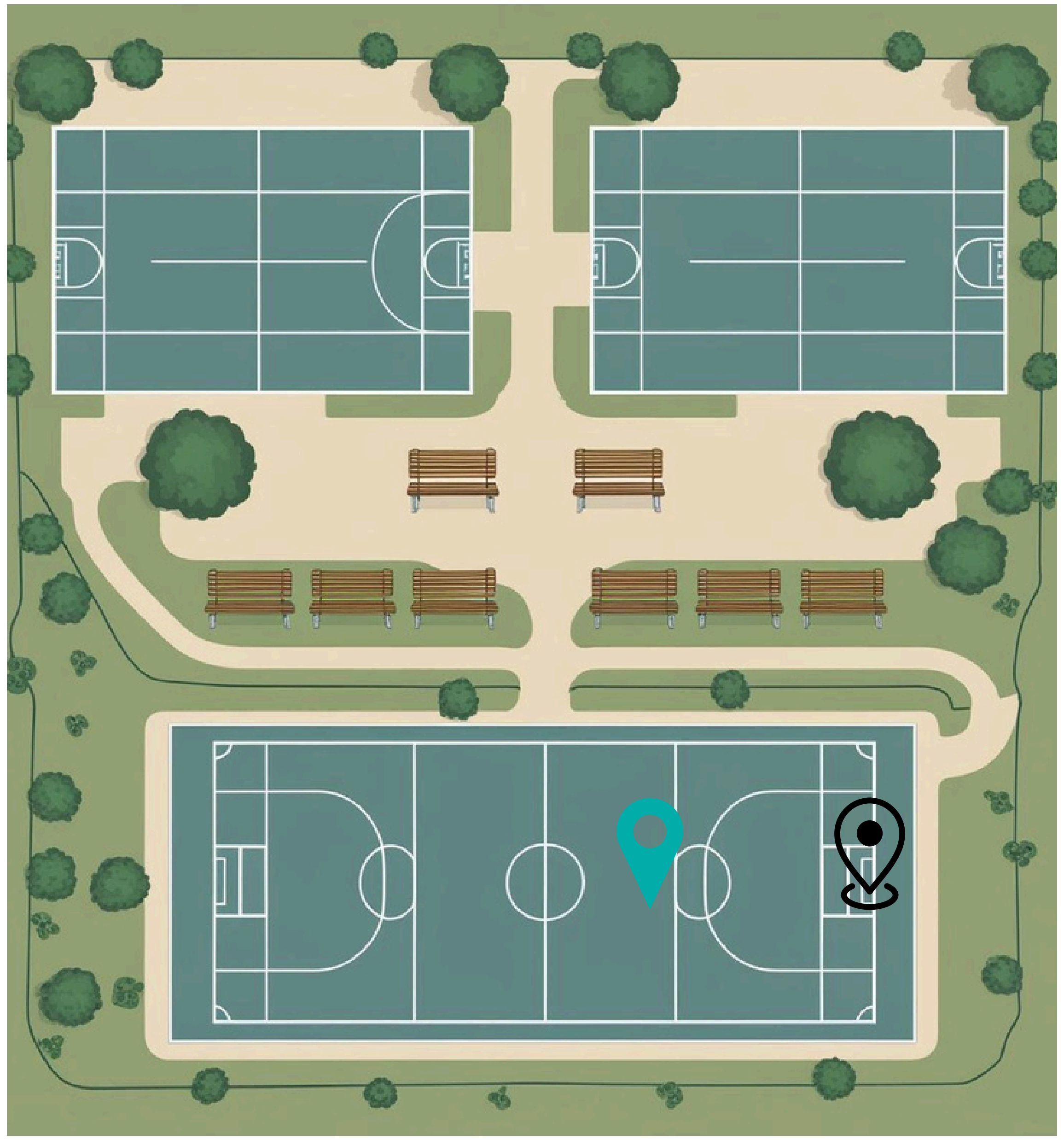

1. In which of these sports do I need to collaborate with my teammates while also facing multiple opponents?
2. Which of the sports shown in the pictures can be watched at the Winter Olimpics?
3. Which of the following sports is individual as well as a combined sport?
4. Which of the following pictures represents a sport involving opponents?
5. Which of the following pictures represents a traditional sport?
6. If I am watching a precision sport, which sport am I watching?

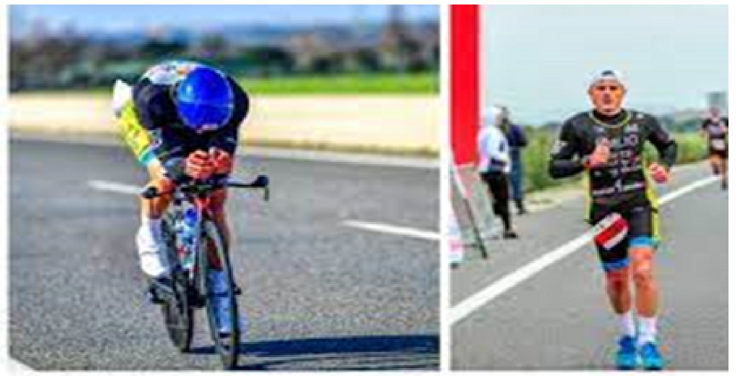

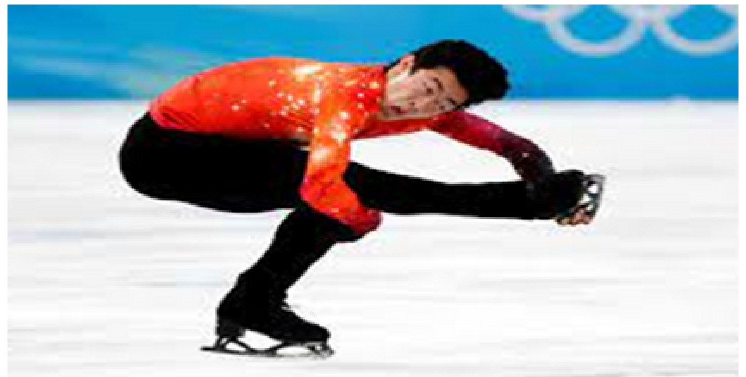

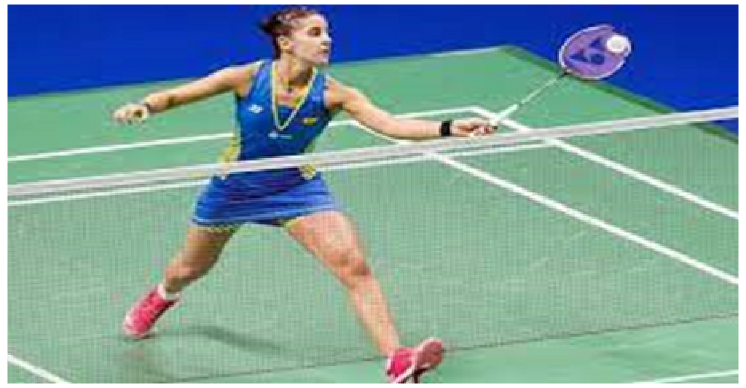

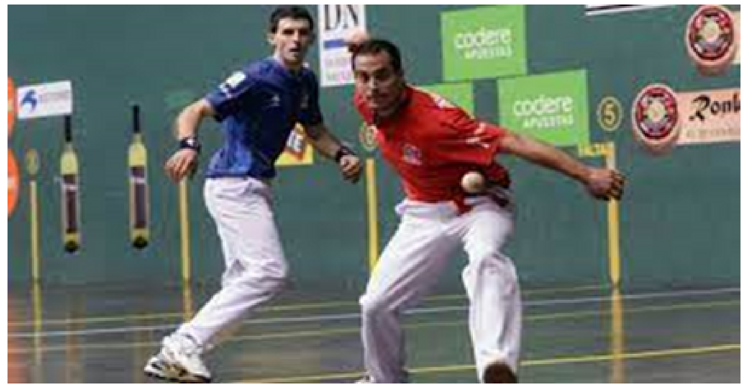

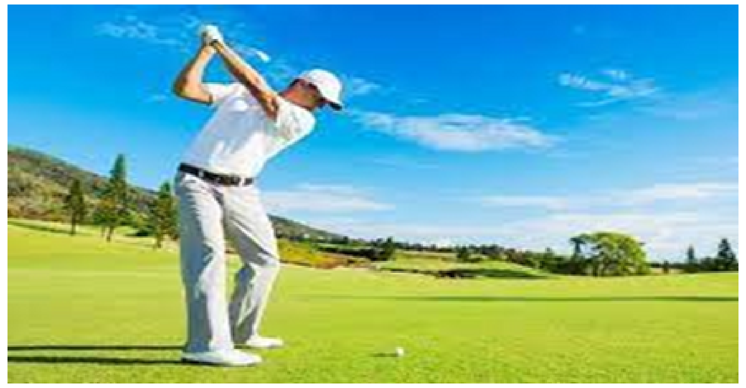

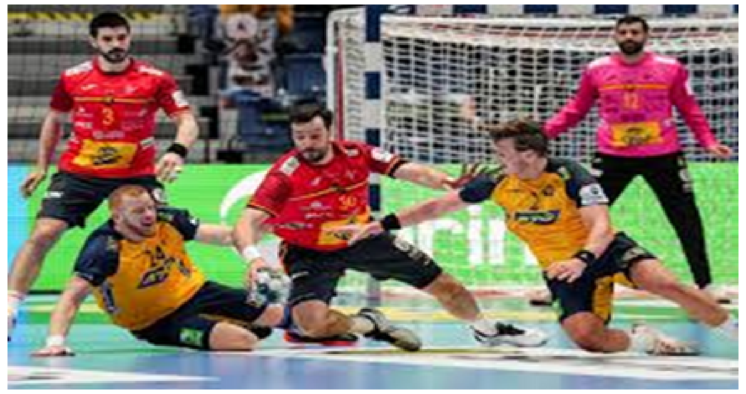

WEEK 6 - SESSION 3

LINK THE ANSWER

Participants are organized into different groups. Each group (represented by a colored marker) has a card with different options, which correspond to the possible answers.

The questions are located at the central point (black marker). A representative from each group must run to the central point to read the question aloud, so it can be shared with the group and aswered collaboratively.

The participant who initially went to read and retrieve the first question for their group, cannot read the next question. Another participant must take their place.

Once all the questions have been answered, a representative from each team will present the answers to the isntructor for verification. If the answers are incorrect, the gorup may modify them and attempt to complete the activity correctly. The team that answers all the questions correctly first win.

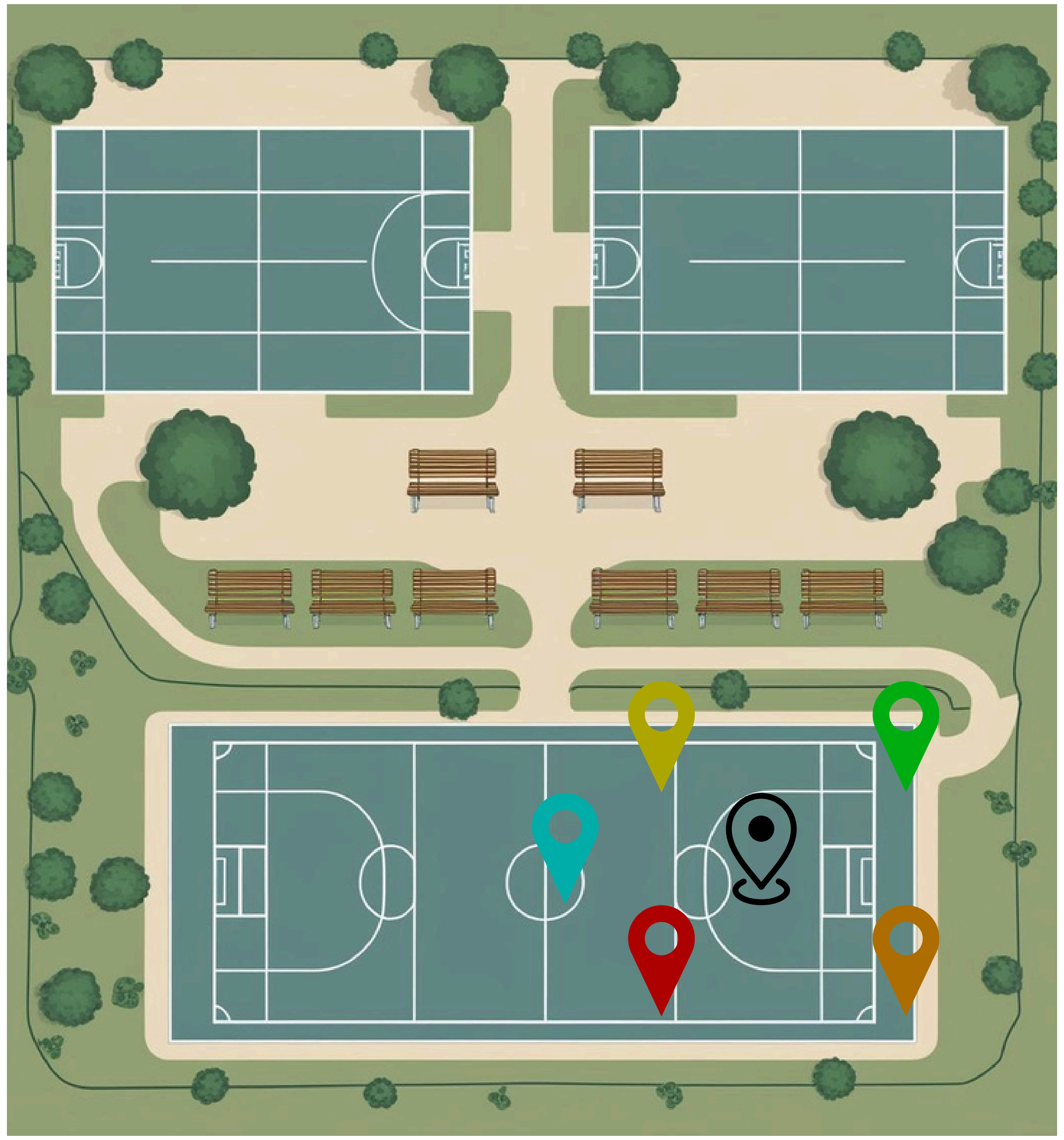

1. By increasing my strength, I will improve my...
2. If I practice an endurance sport such as rowing, one of the benefits I will get for my body will be...
3. If I want to run a marathon, what basic physical capactiy will I have to improve in order to finish the race?
4. If I want to lift a weight off the ground, what basic physical capacity will I mainly use?
5. Write a strength exercise and an endurance exercise.

Group__

ANSWERS

1.

2.

3.

4.

5.

OPTIONS

a) Strenght

b) Quality of life

c) Resistance

d) Flexibility

e) Strengthen my heart
